# Targeting Fatty Acid Binding Protein (FABP) Anandamide Transporters – A Novel Strategy for Development of Anti-Inflammatory and Anti-Nociceptive Drugs

**DOI:** 10.1371/journal.pone.0050968

**Published:** 2012-12-07

**Authors:** William T. Berger, Brian P. Ralph, Martin Kaczocha, Jing Sun, Trent E. Balius, Robert C. Rizzo, Samir Haj-Dahmane, Iwao Ojima, Dale G. Deutsch

**Affiliations:** 1 Biochemistry and Cell Biology, Stony Brook University, Stony Brook, New York, United States of America; 2 Applied Mathematics and Statistics, Stony Brook University, Stony Brook, New York, United States of America; 3 Chemistry and the Institute of Chemical Biology and Drug Discovery, Stony Brook University, Stony Brook, New York, United States of America; 4 Research Institute on Addictions, University at Buffalo, Buffalo, New York, United States of America; University of Milan, Italy

## Abstract

Fatty acid binding proteins (FABPs), in particular FABP5 and FABP7, have recently been identified by us as intracellular transporters for the endocannabinoid anandamide (AEA). Furthermore, animal studies by others have shown that elevated levels of endocannabinoids resulted in beneficial pharmacological effects on stress, pain and inflammation and also ameliorate the effects of drug withdrawal. Based on these observations, we hypothesized that FABP5 and FABP7 would provide excellent pharmacological targets. Thus, we performed a virtual screening of over one million compounds using DOCK and employed a novel footprint similarity scoring function to identify lead compounds with binding profiles similar to oleic acid, a natural FABP substrate. Forty-eight compounds were purchased based on their footprint similarity scores (FPS) and assayed for biological activity against purified human FABP5 employing a fluorescent displacement-binding assay. Four compounds were found to exhibit approximately 50% inhibition or greater at 10 µM, as good as or better inhibitors of FABP5 than BMS309403, a commercially available inhibitor. The most potent inhibitor, γ-truxillic acid 1-naphthyl ester (ChemDiv 8009-2334), was determined to have K_i_ value of 1.19±0.01 µM. Accordingly a novel α-truxillic acid 1-naphthyl mono-ester (SB-FI-26) was synthesized and assayed for its inhibitory activity against FABP5, wherein SB-FI-26 exhibited strong binding (K_i_ 0.93±0.08 µM). Additionally, we found SB-FI-26 to act as a potent anti-nociceptive agent with mild anti-inflammatory activity in mice, which strongly supports our hypothesis that the inhibition of FABPs and subsequent elevation of anandamide is a promising new approach to drug discovery. Truxillic acids and their derivatives were also shown by others to have anti-inflammatory and anti-nociceptive effects in mice and to be the active component of Chinese a herbal medicine (*Incarvillea sinensis*) used to treat rheumatism and pain in humans. Our results provide a likely mechanism by which these compounds exert their effects.

## Introduction

**Figure 1:**
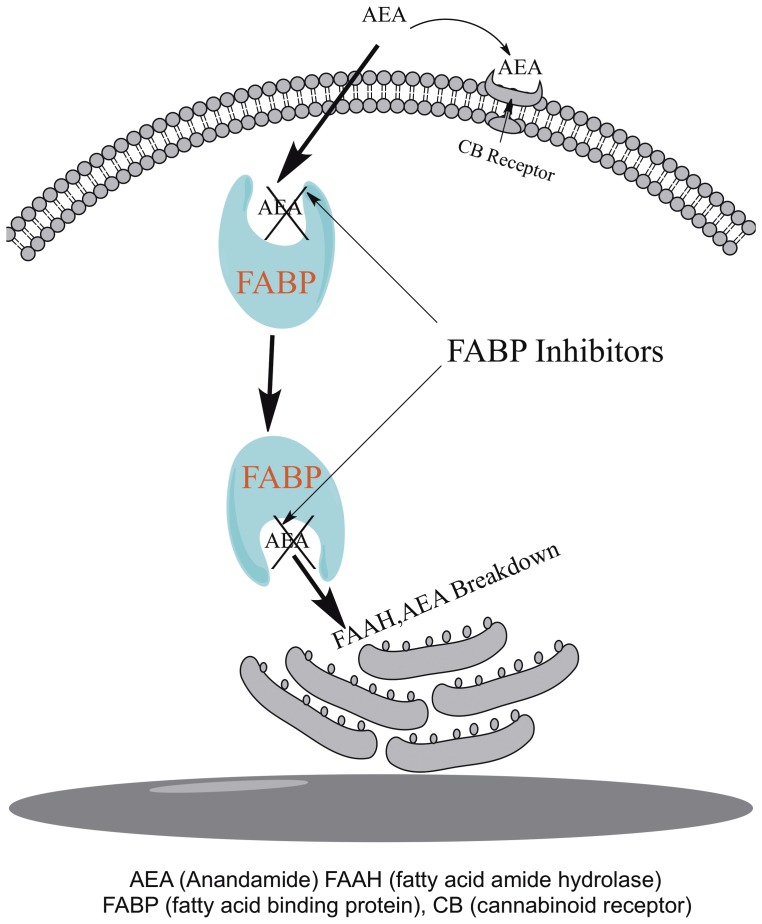
Scheme demonstrating anandamide inactivation and FABP drug target. Anandamide crosses the membrane by diffusion but requires FABPs for transport through the cytoplasm to the endoplasmic reticulum for breakdown by FAAH. FABP inhibitors prevent AEA from being delivered to FAAH for breakdown resulting in increased AEA levels at the receptor.

Lipids, owing much to their water insolubility, require a variety of fatty acid binding protein (FABP) chaperones or transporters to carry them throughout cells [1], [2]. Recently, it was shown that anandamide (an endocannabinoid) also uses FABPs, such as FABP5 (E-FABP) and FABP7 (B-FABP), as intracellular transporters [2]. Thus, FABPs have been recently found to serve a critical part in the pathway for anandamide inactivation by fatty acid amide hydrolase (FAAH), an enzyme localized inside the cell on the endoplasmic reticulum (Figure 1). FABPs are drug targets similar to FAAH in that inhibitors of each decrease hydrolysis of anandamide and its uptake into cells, raising levels of extracellular anandamide, a ligand that targets cannabinoid (CB) receptors [3], [4], [5]. However, unlike FAAH that is distributed throughout the body, there are approximately ten human FABPs with appreciable tissue specificity. For example, FABP3 (heart FABP), FABP5 (epidermal FABP), FABP7 (brain FABP) and FABP8 (myelin FABP) are all expressed in nervous and other tissues. FABP1 (liver FABP) and FABP4 (adipose FABP), as their names imply, are abundantly expressed in the liver and adipose tissue [6].

Few specific FABP inhibitors have been described. There are those specifically designed for FABP4 (adipocyte FABP), such as BMS309403, important for the protective effects that they exert in several metabolic syndromes and atherosclerosis [7], [8]. We have shown that BMS309403 also binds FABPs, such as FABP5 and FABP7 that carry anandamide as do other inhibitors, originally designed to inhibit a putative anandamide transmembrane transporter [4]. In an attempt to identify new FABP inhibitors, in this work, we have conducted a large-scale computational virtual screen of over one million commercially available compounds and purchased a subset for subsequent experimental evaluation. High-throughput virtual screening is a powerful and practical approach for screening ligand libraries to identify new drug-like leads.

**Figure 2:**
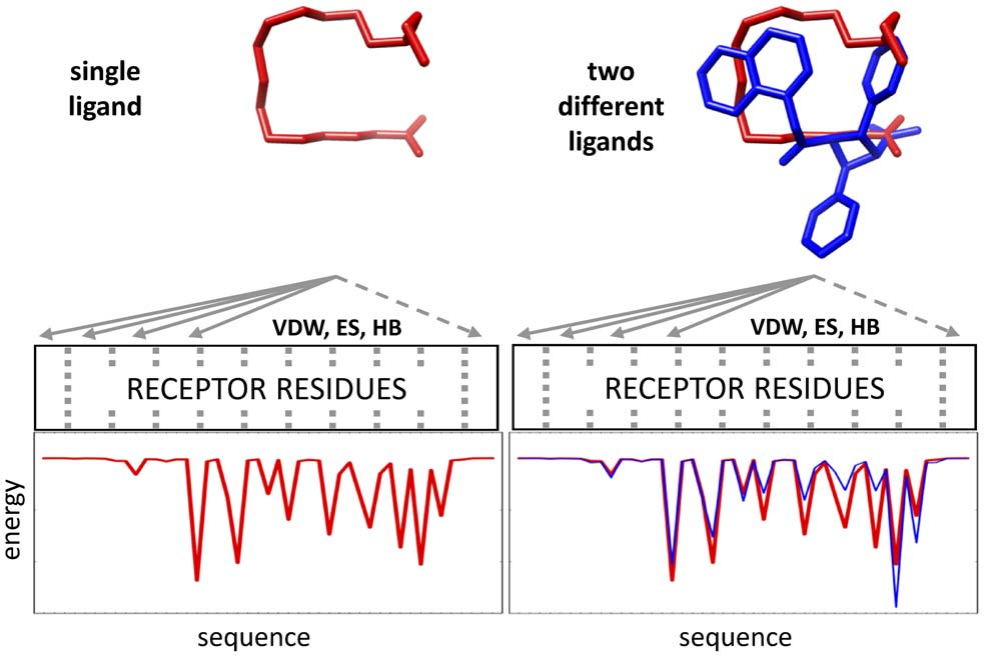
Use of molecular footprint comparisons to prioritize compounds from virtual screening. In this example the van der Waals footprint made by the reference ligand oleic acid (red) is compared with that of a candidate molecule (blue) from the virtual screen.

Traditionally, many screening programs such as DOCK [9], employ a simple two-term scoring function (score) consisting of intermolecular van der Waals and electrostatic terms to rank-order compatibility of ligands with a target. In this work, we utilize a new scoring function termed molecular footprint similarity (FPS) score [10] in which the standard DOCK energy score is decomposed into per-residue contributions. The method was recently employed in the successful discovery of novel HIVgp41 inhibitors [11]. As illustrated in Figure 2, the procedure can be used to identify which compounds are most energetically similar to a known reference. In this example, the van der Waals interaction pattern made by the natural FAPB substrate oleic acid (red lines) is compared with that of a candidate ligand (blue line). The hypothesis is molecular footprints will enrich for active compounds (positives) by facilitating identification of compound making interaction signatures similar to known binders.

Specific goals of this research include: (1) Screen approximately one million compounds using a subset of the ZINC [12] database (ChemDiv. vendor, http://us.chemdiv.com/) of commercially available compounds to FABP7 to prioritize compounds for purchase. (2) Experimentally evaluate purchased compounds in a fluorescence displacement assay to determine binding activity. (3) Determine which hits are most promising for further development. Given the potential beneficial effects on stress, pain, and inflammation continued identification and development of new inhibitors of FAPBs is important.

## Results and Discussion

### High-throughput virtual screening

**Figure 3:**
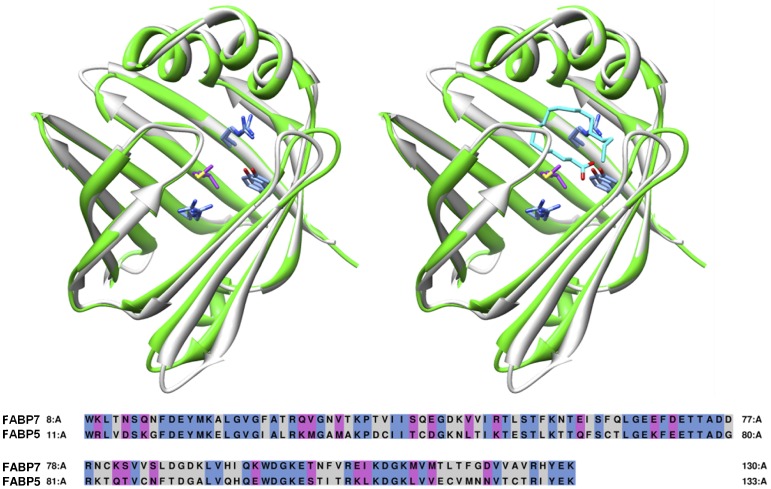
Sequence and structural alignment of FABP7 (grey) with FABP5 (green). Key binding residues consisting of ARG106, ARG126, and TYR128 are identical (blue) however MET115 in FABP7 is substituted for VAL115 in FABP5 (purple). The native substrate oleic acid is shown in cyan.

The CB-1 receptor is predominately expressed in the brain and thus both FABP5 and FABP7 were considered relevant targets for our virtual screening. FABP5 or epidermal fatty acid binding protein (E-FABP) is typical dispersed throughout the body (tongue, adipose tissue, dendritic cell, mammary gland, brain neurons, kidney, liver, lung and testis) and found abundantly in the epidermal cells of the skin. FABP7 or brain fatty acid binding protein (B-FABP) is typically expressed in high levels during mid-term embryonic development but not present in neurons. Structural alignment (0.93 Å RMSD) of FABP7 (PDB: 1FE3, 2.8 Å resolution) and FABP5 (PDB: 1B56, 2.05 Å resolution) revealed a 47% sequence identity and 66% similarity (Figure 3). Furthermore, both FABP7 and FABP5 bind fatty acid substrates with high affinity; although FABP7 typically shows higher binding affinity *in-vitro*. Thus, FABP7 was selected as our initial target for virtual screening.

**Figure 4:**
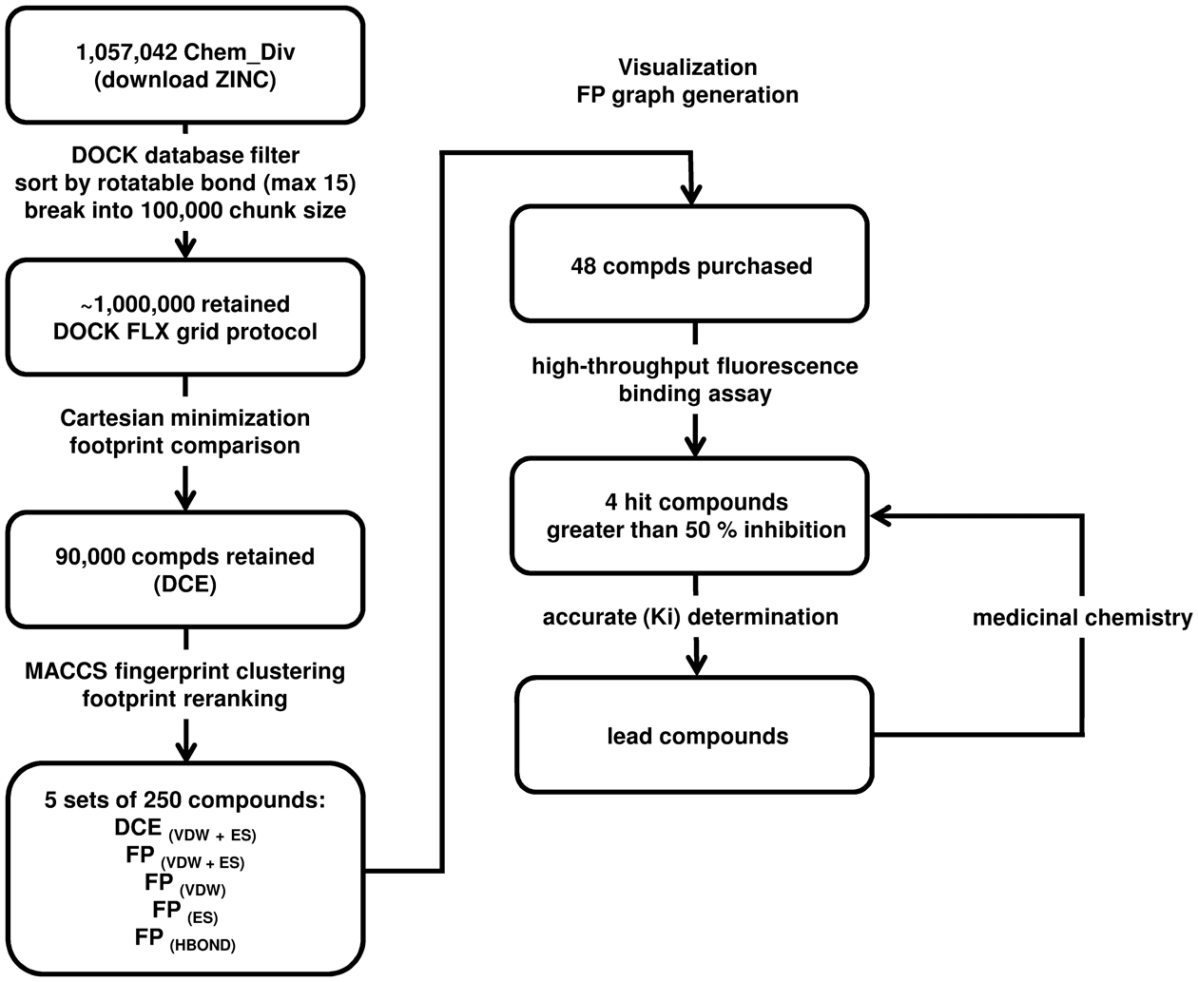
A flow chart describing the discovery of ligands through virtual screening, biological assay, and medicinal chemistry.

High-throughput virtual screening utilizing molecular footprints was conducted on FABP7 using oleic acid as the reference molecule. This entailed: 1) grid setup and docking 2) minimization of each docked molecule and reference molecule on the Cartesian coordinates, 3) calculating the molecular footprints of all docked molecules and reference, 4) calculation of a footprint similarity score for each of the docked molecules versus the reference oleic acid, 5) fingerprint clustering, 6) rank-ordering based on each scoring criteria, 7) analysis and selection of compounds from each of the 250 cluster heads generated for each of the scoring criteria using visual inspection of binding poses and footprints. Figure 4 summarizes the overall work flow.

**Figure 5:**
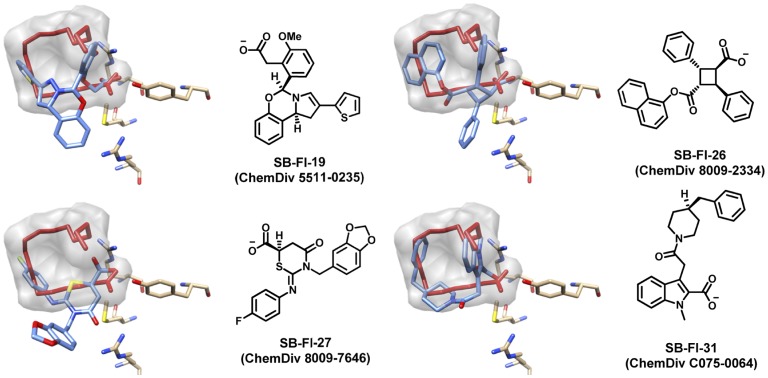
Four compounds from the FABP7 computational virtual screen which show experimental activity in an FABP5 fluorescence displacement assay. The predicted binding pose for each ligand (blue stick) is shown in relationship to the reference oleic acid (red stick, gray surface).

**Figure 6:**
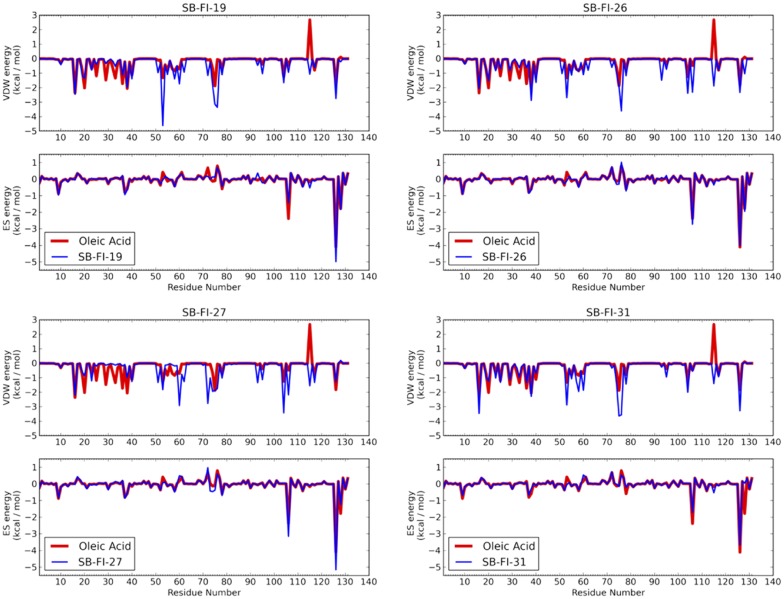
VDW (top) and ES (bottom) footprints for active compounds (blue) compared with the native substrate oleic acid (red). The VDW clash between oleic acid and MET115 is offset by the strong favorable ES interactions at ARG106, ARG126, and TYR 128.

As a result of the virtual screening to FABP7, 48 compounds were purchased and subsequently assayed *in-vitro* against FABP5. The FABP5 homolog was chosen for the initial experimental testing owing to ease of experimental expression compared to FAPB7. Notably, out of the 48 compounds tested, 23 showed at least 25% inhibition and 4 compounds, having the following ChemDiv ID (Stony Brook ID) numbers 5511-0235 (SB-FI-19), 8009-2334 (SB-FI-26 or SB-FI-49), 8009-7646 (SB-FI-27), and C075-0064 (SB-FI-31) showed approximately 50% inhibition or greater (see next section). It is important to note that the isomer provided by the vendor for ChemDiv ID 8009-7646 was the gamma form that is in contrast to alpha isomer downloaded from the ZINC database which was docked into FABP7. The alpha form however was synthesized latter (see Methods), and experimentally evaluated, although not in the initial fluorescence displacement assays. To avoid confusion and throughout this manuscript, the actual isomer that was used in any given computational or experimental test is indicated as either SB-FI-26 (α-isomer) or SB-FI-49 (γ-isomer). Figure 5 shows the predicted DOCK binding pose for the four active compounds in relationship to the reference oleic acid. Figure 6 shows the accompanying van der Waals and electrostatic footprint overlaps. Table 1 shows numerical values for the DOCK and FPS scores.

**Table 1:** Dock energy and footprint similarity (FPS) scores for compounds docked to FABP7.

Compound ID^a^	Method^b^	DCE_VDW+ES_ c	FPS_VDW+ES_ d	FPS_VDW_ d	FPS_ES_ d
SB-FI-195511-0235	FPS_VDW+ES_	−56.56	1.09	0.81	0.28
SB-FI-268009-7646	FPS_VDW+ES_	−56.76	1.01	0.87	0.14
SB-FI-278009-7646	FPS_ES_	−53.34	1.21	1.00	0.21
SB-FI-31C075-0064	FPS_VDW+ES_	−53.77	1.12	0.73	0.39

a Stony Brook ID with corresponding ChemDiv number.

b Footprint scoring function used for compound selection.

c DCE scores in kcal/mol.

d FPS scores in units of normalised Euclidian distance where 0 represents the best overlap. FPS_VDW_ and FPS_ES_ scores range from (0,2] and FPS_VDW+ES_ range from (0,4].

The determining factor in compound selection was use of the FPS scoring function thus it is expected that the four hit compounds will have overlap with the oleic acid reference. As shown in Figure 6, and quantified numerically in Table 1, all actives have substantial overlap in ES footprints (FPS_ES_ scores from 0.14 to 0.39) and to a lesser extent VDW footprints (FPS_VDW_ scores from 0.73 to 1.00). Interestingly the overlays in Figure 5 show that three inhibitors (SB-FI-19, and SB-FI-27) spatially deviate from the surface defined by the reference which likely accounts for their poorer FPS_VDW_ scores (0.81 to 1.00) compared to SB-FI-31 (0.73). However, SB-FI-31 also has the poorest ES overlap (0.39) among the four thus it is not the most favorably scored compound overall. Importantly, all four actives contain a charged carboxylate moiety, analogous to that in the reference oleic acid (Figure 5), which predicted to occupy the same position in the FAPB binding site. All make strong ES interactions with ARG106, ARG126, and in particular at position TYR128 (Figure 5, Figure 6). The footprints indicate that SB-FI-19 and SB-FI-26 interact somewhat more strongly with TYR128 compared with SB-FI-27 and SB-FI-31 which could play a role in these compounds being the two most potent inhibitors among the four identified (see experimental results). Interestingly, SB-FI-19 and SB-FI-26 also have the most favorable DCE_VDW+ES_ scores among the group.

**Figure 7:**
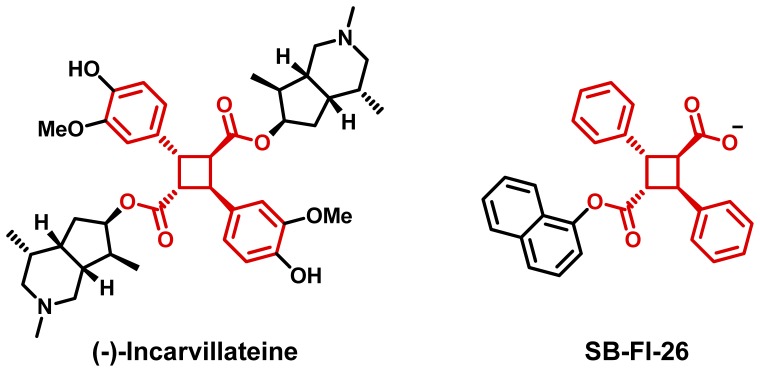
Highlighted in red is the α-truxillic acid core structure of both (-)-Incarvillateine and FABP inhibitor SB-FI-26.

Overall, based on potency and taken into consideration its scaffold, SB-FI-26 was selected for further exploration and development. The core of SB-FI-26 is a prominent structural component of the natural product incarvillateine which is known to produce graded inhibition of pain and inflammation in formalin-induced mouse models, although the mechanism of action was not fully understood (Figure 7) [13]. Furthermore, structural exploration of α-truxillic analogues and various α-truxillic acid di-esters derivatives of the natural product were investigated, producing similar effects in mouse models [14], [15]. Thus, SB-FI-26 provided a means to probe mechanisms underlining the viability of using α-truxillic acid based mono-esters and in particular the 1-naphthyl ester derivative to treat pain and inflammation.

### Identification of Lead Compounds – Fluorescence Displacement Assay

**Figure 8:**
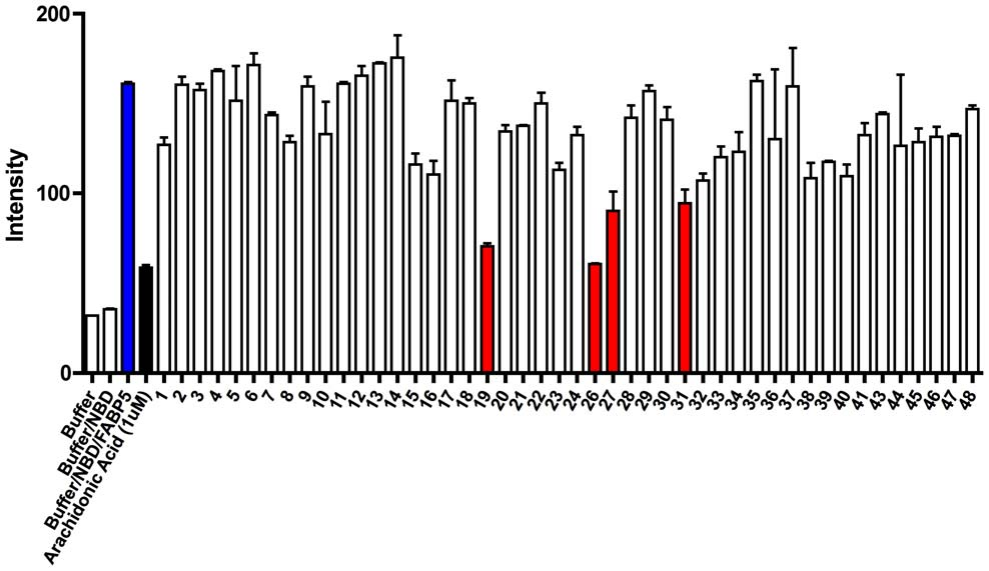
High throughput fluorescence displacement assay with NBD-stearate. Shown in blue is the NBD-stearate FABP5 complex with no inhibitor, in black is arachidonic acid a potent inhibitor of FABP5, and in red are the four lead compounds.

The initial experimental binding evaluation of the 48 compounds purchased from the virtual screen utilized an established fluorescence displacement assay. The degree to which the 48 test compounds (10 µM) displaced NBD-stearate (1 µM) from FABPs is shown in Figure 8. The first two samples, the buffer and NBD-stearate do not give appreciable fluorescence while the NBD-stearate plus purified FABP5 gives an appreciable fluorescence signal (blue bar). The forth sample is the positive control where arachidonic acid (1 µM), a fatty acid that binds strongly to FABP5 (Ki, 0.13 µM) decreases the signal. Each sample in this screen was measured in duplicate and approximately 1/3 of the test compounds appeared to cause displacement of NBD-stearate with a concomitant decrease in fluorescence. Four of the most potent (red bars) were selected for further evaluation and statistical analysis.

**Figure 9:**
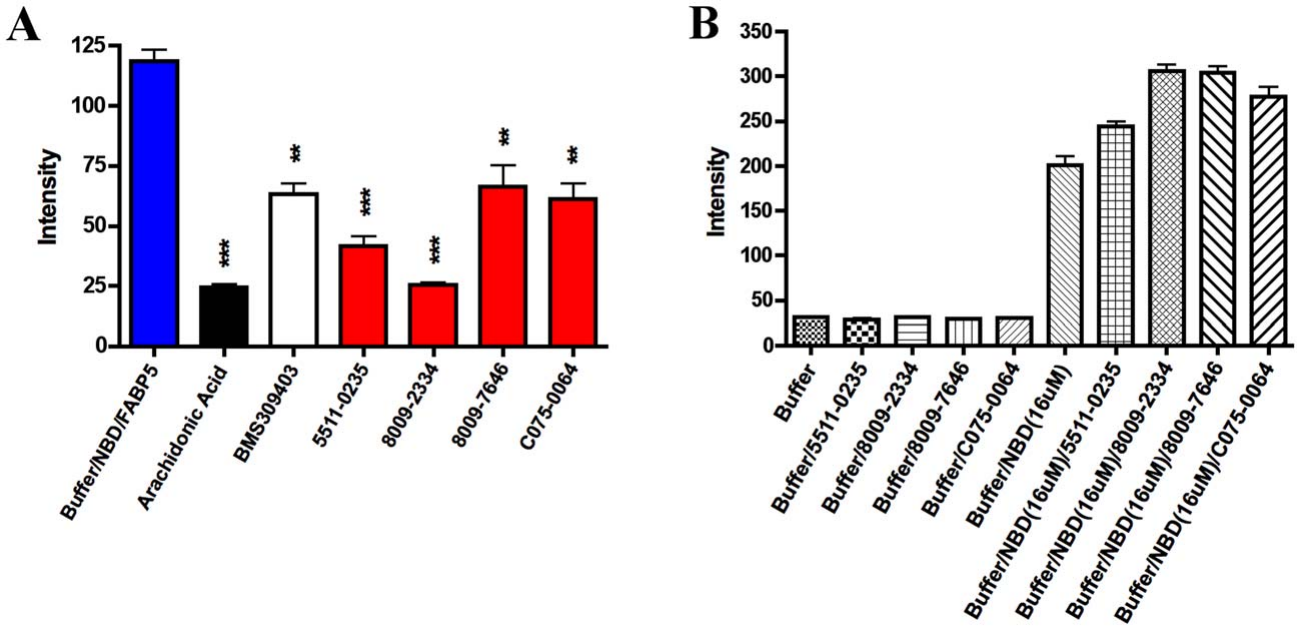
Verification of high throughput fluorescent displacement assay results. (A) Replicate testing of the lead compounds shows that 8009-2334 (SB-FI-26) exhibits the best inhibition of FABP5. One * indicates p<0.05, two ** indicates p<0.01, three *** indicates p<0.001 (n = 3). (B) Controls show that all lead compounds exhibited no detectable fluorescence in the assay nor did they add significantly to the fluorescence of the NBD-stearate probe.

Of all chemical compounds virtually screened (Figure 8), compounds 5511-0235 (19), 8009-2334 (26), 8009-7646 (27), and C075-0064 (31) proved to have the strongest interactions with FABP binding site in both virtual and biological screening. These compounds were then rerun in the NBD-fluorescent assay with replicate measurements at 10 µM. As shown in Figure 9A, inhibition of NBD-stearate binding to FABP5 by these compounds was highly significant with ChemDiv 8009-2334 (26) the most potent. In control experiments, it was observed that the four lead compounds did not fluoresce under the assay conditions at 10 µM, nor did 10 µM of these compounds quench the fluorescence of 16 µM NBD-stearate, that is 16 times the fluorophore concentration used in the routine assay (Figure 9B).

**Figure 10:**
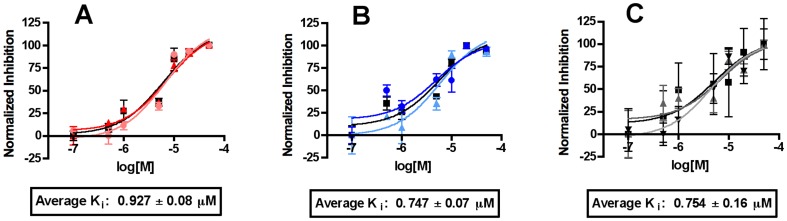
Binding analysis of SB-FI-26 (α-truxillic acid 1-naphthyl ester), SB-FI-49 (γ-truxillic acid 1-naphthyl ester), and BMS309403. (A) Assay in triplicate shows that SB-FI-26 derivative attains a K_i_ within nanoMolar ranges. (B) Analysis of γ-truxillic acid 1-naphthyl ester implies that 8009-2334 is an impure form of the compound and that the pure, synthesized gamma derivative is as potent as BMS309403. (C) BMS309403 was found to be slightly more potent in this study (K_i_, 0.75 µM) than published (K_i_, 0.89 µM), but still within range of this value.

As noted above, the virtual screening of ChemDiv ID 8009-2334 employed α-truxillic acid 1-naphthyl mono-ester (SB-FI-26) however the form supplied by the vendor and thus experimentally evaluated, was γ-truxillic acid 1-naphthyl mono-ester (SB-FI-49). To reconcile the differences as well as provide additional quantities both α- and γ- forms were chemically synthesized in-house (see Methods). Importantly, the ^1^H NMR spectrum of the newly synthesized α-form (SB-FI-26) did not match that of the ChemDiv 8009-2334 but the newly synthesized γ-form (SB-FI-49) did, thus confirming the isomeric form that was provided. The inhibitory activity of the newly synthesized alpha form SB-FI-26 was assayed and a K_i_ value of 0.93±0.08 µM was determined from triplicate analysis (Figure 10A). The inhibitor activity of the newly synthesized gamma form SB-FI-49 led to an improved Ki value of 0.75±0.07 µM, which was higher than that from the vendor sample (1.19±0.01 µM) presumably due to greater purity.

Overall, SB-FI-49 at a Ki value of 0.75±0.07 µM appears equally as potent as BMS309403 (K_i_ 0.75±0.16 µM) as shown in Figure 10C. However, although SB-FI-49 appeared to be the most potent inhibitor identified in this study it was substantially less soluble (200 µM in DMSO at 25°C) than either the BMS309403 (1 mM in DMSO at 25°C), or surprisingly, the alpha form SB-FI-26 (1 mM in DMSO at 25°C). Thus, SB-FI-26 provided to be the best lead compound for further evaluation.

The two most potent compounds, SB-FI-26 and SB-FI-49 (the α-and γ-truxillic acid 1-naphthyl esters), discerned from our *in silico* and biological screening, belong to a class of compounds that have been found to have anti-inflammatory and anti-nociceptive [14], [15]. Heretofore, the mechanism by which these effects were mediated was unknown. However, we can speculate that these compounds inhibit the transport of anandamide and other fatty acid ethanolamides, such as palmitoylethanolamide and oleoylethanolamide. These increased NAE levels would lead to greater signaling at the cannabinoid and potentiate NAE-mediated hypoalgesic and anti-inflammatory effects, indicating that modulation of NAE signaling may represent a therapeutic avenue for the treatment of pain.

### Effects of SB-FI-26 on CB1, PPARα and PPARγ

**Figure 11:**
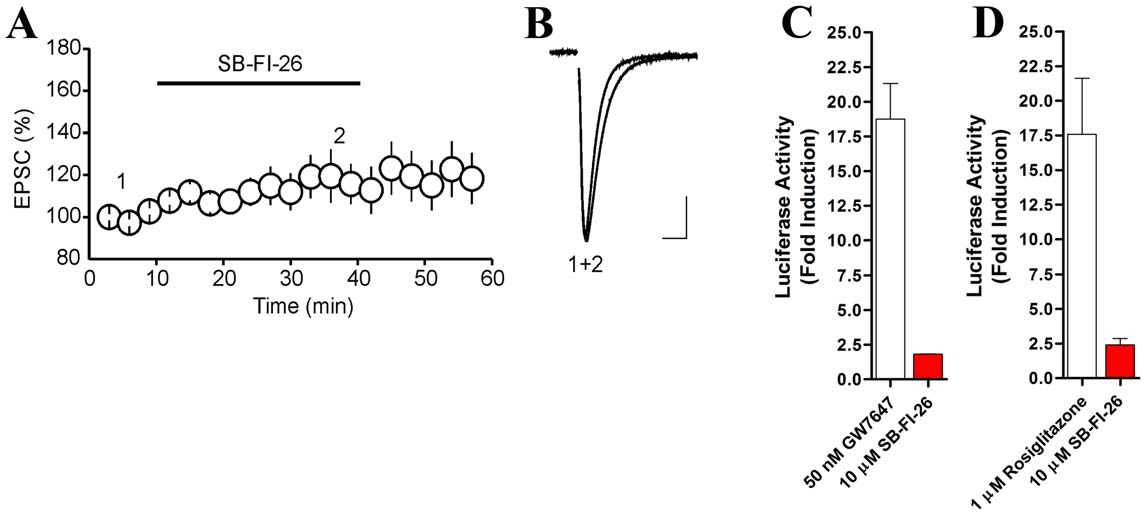
FABP inhibitor SB-FI-26 does not reduce amplitude of EPSCs and is a weak agonist at PPARα and PPARγ receptors. (A) Summary graph of effect of SB-FI-26 (10 µM) on amplitude of EPSCs. (B) Superimposed average EPSC traces taken at the time point indicated by numbers in panel A. Note that application of α-1-napthol-truxillic acid did not inhibit the amplitude of EPSCs. (C) PPARα activation by SB-FI-26 and the PPARα agonist GW7647. (D) Activation of PPARγ receptors by SB-FI-26 compared to the PPARγ agonist rosiglitazone (n = 3).

Activation of cannabinoid CB1 receptors inhibits glutamatergic synaptic transmission in numerous brain areas, including the dorsal root ganglion DR [16]. Therefore, to test whether SB-FI-26 exhibits agonist properties at CB1 receptors, we examined its effects on the amplitude of glutamate-mediated excitatory postsynaptic currents (EPSCs) recorded from DR 5-HT neurons. We found that bath application of SB-FI-26 (10 µM) did not inhibit the amplitude of EPSCs (107±6.7% of baseline, n = 8, Figure 11A, 11B). Such a finding suggests that SB-FI-26 is not a CB1 receptor agonist.

Previous reports indicate that α-truxillic acid derivatives activate peroxisome proliferator-activated receptor γ (PPARγ), which alongside PPARα, modulate nociception [17], [18], [19]. In our hands, SB-FI-26 served as a weak agonist at both receptors, displaying ∼2-fold activation of PPARα and ∼3-fold activation of PPARγ (Figure 11C, 11D).

### Effect of SB-FI-26 upon AEA uptake in cells

**Figure 12:**
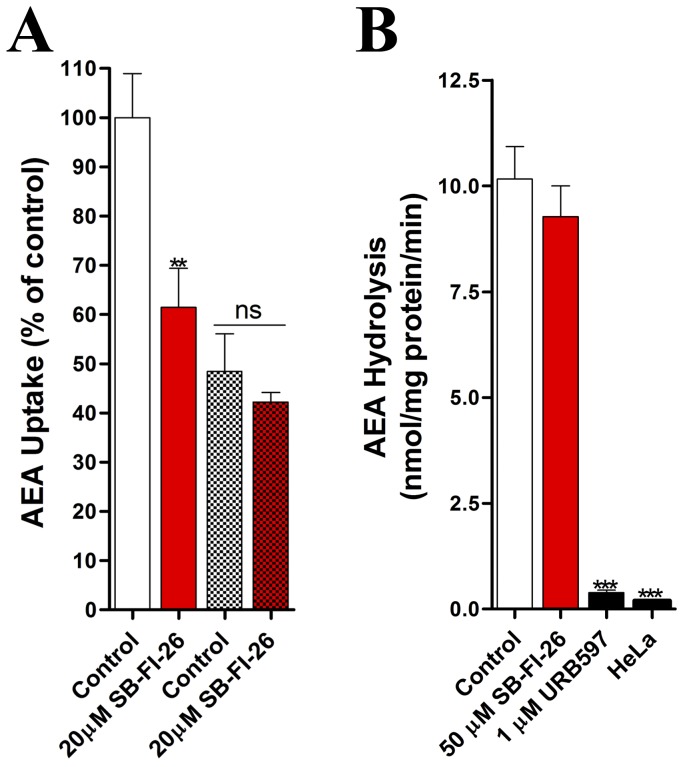
SB-FI-26 inhibits the cellular uptake of AEA. (A) AEA uptake in wild-type HeLa (un-shaded) or FABP5 shRNA-expressing HeLa (shaded) cells in the presence or absence of SB-FI-26. (B) AEA hydrolysis in FAAH-transfected HeLa cells in the presence or absence of SB-FI-26 or the FAAH inhibitor URB597. **, p<0.01; ***, p<0.001 (n = 3).

We have previously shown that FABPs are intracellular carriers that shuttle endocannabinoids and related N-acylethanolamines to intracellular sites, such as FAAH for hydrolysis [2], [4]. Pharmacological or genetic inhibition of FABPs reduces AEA catabolism in cells, confirming an essential role for these proteins in endocannabinoid inactivation [2], [4]. Therefore, we examined whether the novel FABP inhibitor SB-FI-26 reduces FABP-mediated AEA uptake in cells. Indeed, SB-FI-26 significantly inhibited cellular AEA accumulation (Figure 12A). Confirming its selectivity for FABPs, SB-FI-26 failed to reduce AEA uptake in cells bearing a knockdown of FABP5 (Figure 12A), the main FABP expressed in HeLa cells [2], [4]. Additionally, SB-FI-26 does not inhibit FAAH (Figure 12B). Collectively, these results indicate that SB-FI-26 is a selective FABP inhibitor.

### SB-FI-26 produces antinociceptive and anti-inflammatory effects in mice

**Figure 13:**
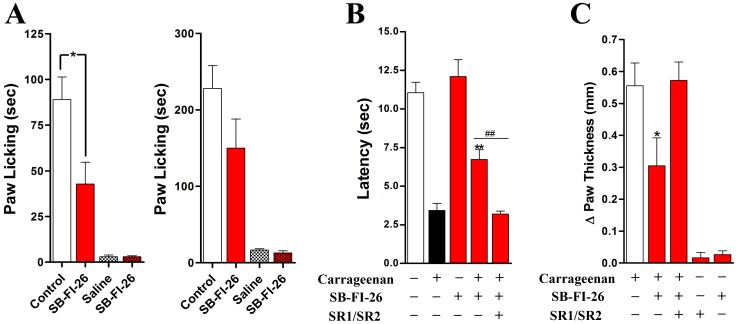
Antinociceptive effects of SB-FI-26. (A) SB-FI-26 (20 mg/kg, i.p.) reduces pain associated with the first phase (left panel) but not the second phase (right panel) of the formalin test. Intraplantar injection of vehicle or 20 µg SB-FI-26 (checkered bars) did not evoke nocifensive behavior. *, p<0.05 (n = 6). (B) SB-FI-26 (20 mg/kg, i.p.) alleviates carrageenan-induced thermal hyperalgesia in mice. Concurrent administration of rimonabant and SR144528 (SR1/SR2) blocked the antinociceptive effects of SB-FI-26. **, p<0.01 versus carrageenan-injected animals; ##, p<0.01 versus SR1/SR2-treated animals (n = 6–9). (C) SB-FI-26 (20 mg/kg, i.p.) reduces carrageenan-induced paw edema. *, p<0.05 (n = 6–9).

Similar to cannabinoid receptor agonists, inhibitors of endocannabinoid inactivation produce anti-inflammatory and antinociceptive effects [20], [21]. Importantly, FAAH inhibitors lack the untoward psychotropic effects of cannabinoid receptor agonists [22], highlighting the therapeutic advantage of pharmacologically targeting endocannabinoid inactivation. Because inhibition of AEA transport to FAAH reduces AEA inactivation, we hypothesized that FABP inhibitors may likewise possess antinociceptive and anti-inflammatory properties. Therefore, we examined the effects of SB-FI-26 using two nociceptive models: the formalin test and carrageenan-induced thermal hyperalgesia. In the formalin test, injection of formalin results in the induction of two temporally distinct phases of pain with the first phase (0–5 min) representing nociceptor activation and the second phase (15–45 min) representing inflammatory pain and central sensitization. As shown in Figure 13A, SB-FI-26 significantly reduced nocifensive behavior only during the first phase of the formalin test and SB-FI-26 did not evoke nocifensive behavior when administered alone.

We next explored whether SB-FI-26 alleviates inflammatory pain induced by intraplantar injection of λ-carrageenan. Indeed, SB-FI-26 (20 mg/kg, i.p.) significantly reduced carrageenan-induced thermal hyperalgesia (Figure 13B) and paw edema (Figure 13C). To establish a cannabinoid receptor-mediated mechanism of action, mice were pretreated with a combination of the cannabinoid receptor 1 and 2 antagonists, rimonabant and SR144528, respectively. The antinociceptive and anti-edematous effects of SB-FI-26 were completely reversed by rimonabant and SR144528 (Figure 13B and C) while SB-FI-26 and rimonabant/SR144528 did not induce paw edema when administered alone (Figure 13C).

### Conclusions

The current study reports the identification and characterization of novel small molecule inhibitors of FABP5, obtained through virtual screening to the homologous receptor FABP7, which were identified using the program DOCK in conjunction with a new footprint-based similarity scoring function. Over one million small molecules were docked and the VDW and ES footprint similarity overlap was computed between the reference substrate oleic acid. Forty-eight molecules were ultimately identified for purchase and subsequently assayed against FABP5 using a high-throughput fluorescent displacement assay. Overall, four compounds from the ChemDiv compound library were identified as potential competitive inhibitors of FABP5. The most potent compound, ChemDiv 8009-2334, was found to possess a diphenyl-cyclobutane core characteristic of the known natural product (-)-incarvillateine.

A novel α-truxillic acid 1-naphthyl mono-ester, SB-FI-26, was synthesized and the FABP5 NBD-stearate displacement assay of this compound showed a sub-micro molar Ki value. The resynthesized γ-form of truxillic acid 1-naphthyl mono-ester (SB-FI-49) also showed sub-micro molar efficacy against FABP5, which was considerably more potent than ChemDiv 8009-2334, probably due to the difference in purity. SB-FI-26 (α-form) and SB-FI-49 (γ-form) were found to be as potent as BMS309403, a well-known FABP inhibitor. Thus with both our virtual and biological screening, truxillic acid mono-esters were identified as a unique class of compounds that target FABPs.

Following biological screening and binding analyses of these inhibitors, we have shown that the novel FABP inhibitor SB-FI-26 produces antinociceptive and anti-inflammatory effects in mice. These findings are in agreement with a previous study demonstrating that some α-truxillic acid derivatives exhibited antinociceptive properties, although the mechanism of action was not identified [15]. Interestingly, incarvillateine, comes from the dried plant Incarvillea sinensis (Jiaohao, Kakko, Cheron, Tougucao) used in Chinese herbal medicine to treat rheumatism and relieve pain. Our studies may now ascribe a mechanism by which this natural product and related truxillic acid compounds may exert their effect in part.

It was subsequently reported that certain derivatives of α-truxillic acid activate PPARγ [17]. Although our work demonstrates that SB-FI-26 behaves as a weak agonist at PPARα and PPARγ, its antinociceptive effects were abolished by cannabinoid receptor antagonists. Therefore, the antinociceptive effects of SB-FI-26 likely resulted from potentiation of endocannabinoid signaling rather than activation of PPAR receptors. Taken together, our results establish FABPs as novel targets for antinociceptive drug development. In addition to the FABP transporters described here, heat shock protein 70, albumin, and a truncated fatty acid amide hydrolase protein have also been reported as intracellular shuttles for AEA [23],[24] and this area has been recently review [25]. Our studies show the potential for the design of even more potent inhibitors that will be selective for individual FABPs.

## Materials and Methods

### High-throughput virtual screening

A high-throughput virtual screening of over one million molecules from the ChemDiv subset of the ZINC database (http://zinc.docking.org) was conducted on New York Blue, an 18 rack IBM Blue Gene/L massively parallel supercomputer located at Brookhaven National Laboratory using DOCK version 6.5 [9]. Prior to docking, the most updated ChemDiv database was downloaded and presorted by rotatable bonds and split into 10 subsets of ∼100,000 molecules using the DOCK database filter. Subsequently, an energy grid for FABP7 (PDB : 1FE3) was generated using the grid program [26]. Then, each molecule was flexibly docked to the FABP7 grid (DOCK FLX protocol) [27] and the single lowest-energy pose was retained.

### Footprint-based rescoring

Following high-throughput virtual screening, the footprint-based rescoring methodology reported by Balius et al [10] was implemented to enrich the library of docked molecules. First, the co-crystalized ligand oleic acid (reference) was minimized on the FABP Cartesian coordinates within the binding pocket. This was implemented using both a hydrogen optimization followed by a weak restrained minimization (restraint of 10 kcal/mol). Following reference minimization, each molecule of the docked library was subsequently minimized in Cartesian space using the restrained minimization protocol. Last, electrostatic, van der Waals, and hydrogen bond footprint similarity scores were computed using normalized Euclidian distance for each molecule docked versus the reference using DOCK 6.5.

### Database Clustering and Compound Selection

First, subsets 1 through 5 and subset 6 through 10 containing ∼500,000 molecules were rank-ordered by the DOCK Cartesian energy (DCE) score. The top 45,000 of each combined subset of 500,000 molecules (∼10% total 90,000 molecules) were then clustered using MACCS fingerprints, as implemented in the program Molecular Operating Environment (201110 ed. Chemical Computing Group, Montreal, QC, Canada, http://www.chemcomp.com/software.htm) with the tanimoto coefficient of 0.75. The resulting cluster heads obtained were then further rank-ordered by: (i) standard DOCK score (DCE_VDW+ES_), (ii) van der Waals footprint similarity score (FPS _VDW_), (iii) electrostatic footprint similarity score (FPS_ES_), (iv) H-bond footprint similarity score (FPS_HB_), (v) the sum of van der Waals and electrostatic footprint similarity score (FPS_VDW+ES_). The top 250 molecules rank-ordered by each criteria were then plotted in MathPlotLib [28] and examined by visual inspection and consistency to the reference footprint. This method of analysis allowed us to both visually see key interactions within the binding pocket while simultaneously observing the magnitudes of those key interactions within the footprints for each molecule. Based on this approach, 48 compounds were selected a purchased for biological testing against FABP5. Biological screening of these compounds for activity against FABP7 is underway.

### Chemical Synthesis

#### α-Truxillic acid

*E*-cinnamic acid (1.272 g, 8.59 mmol) was placed in a pyrex dish and exposed to light at 350 nm and an intensity of 280 mW/cm^2^ for 1 week with periodic shaking. This process was performed in the solid state and monitored by ^1^H NMR. After completion of the photoreaction, the white solid was washed with diethylether (200 mL) and allowed to dry. The solid was then recrystallized from pure ethanol to give α-truxillic acid (1.006 g, 79% yield) as a white solid: mp 276–277°C (lit. [29] 274–278°C); ^1^H NMR (300 MHz, DMSO-*d_6_*) δ 12.12 (s, 2H), 7.32 (m, 8H), 7.24 (m, 2H), 4.28 (dd, *J* = 7.2 Hz, 10.1 Hz, 2H) 3.81 (dd, *J* = 7.2 Hz, 10.1 Hz, 2H); ^13^C NMR (75 MHz, DMSO-*d_6_*) δ 173.00, 139.47, 128.19, 127.67, 126.69, 46.17, 41.06. Data are consistent with the literature values [29].

#### α-Truxillic acid 1-naphthyl ester (SB-FI-26)

To 500 mg of pure α-truxillic acid as added 20 mL of tetrahydrofuran (THF) was added along with 1.3 mL of oxalyl chloride. Addition of a drop of *N,N*-dimethylformamide (DMF) allowed conversion of α-truxillic acid to the corresponding diacid dichloride, which was used crude in the subsequent reaction. To α-truxillic acid dichloride, thus prepared, 20 mL of THF was added, followed by 0.7 mL of triethylamine and 100 mg of 4-*N,N*-dimethylaminopyridine(DMAP). To this mixture was added, dropwise, 170 mg of 1-napthol in 10 mL of THF with stirring. The reaction mixture was allowed to stir overnight and quenched with 1M HCl upon completion of the reaction. The reaction mixture was extracted with 100 mL of dichlorormethane and washed with 100 mL of brine. The organic layer was dried over anhydrous MgSO_4_ and concentrated *in-vacuo* to give a brownish oil, which was then purified by flash chromatography on silica gel using 20% ethyl acetate in hexanes followed by 30% ethyl acetate in hexanes as eluents to give α-truxillic acid 1-naphthyl ester (SB-FI-26, 210 mg, 42% yield) as a white solid: mp 195°C; ^1^H NMR (500 MHz, DMSO-*d_6_*) δ 12.24 (s, 1H), 7.88 (d, *J* = 8.5 Hz, 1H), 7.73 (d, *J* = 8.5 Hz, 1H), 7.57 (d, *J* = 7.0 Hz, 2H), 7.52 (d, *J* = 7.5 Hz, 2H), 7.48–7.37 (m, 6 H), 7.34–7.27 (m, 3 H), 7.06 (d, *J* = 8.0 Hz, 1H), 6.38 (d, *J* = 7.5 Hz, 1H) 4.61–4.49 (m, 3H), 3.98 (dd, *J* = 7.0 Hz *J* = 10.0 Hz, 1H); ^13^C NMR (75 MHz, DMSO-*d_6_*) δ 172.73, 170.93, 146.17, 139.23, 139.03, 133.92, 128.74, 128.25, 128.19, 127.89, 127.67, 127.40, 126.90, 126.47, 126.22, 125.85, 125.45, 121.13, 117.86, 46.17, 41.54, 41.11. HRMS (ESI) *m/e* calculated for C_28_H_23_O_4_H^+^: 423.1589. Found: 423.1596 (Δ = 1.7 ppm).

#### 6,7-Diphenyl-3-oxabicyclo[3.1.1]heptane-2,4-dione (γ-truxillic anhydride)

A 20 mL round bottomed flask was charged with a mixture of α-truxillic acid (600 mg, 2.02 mmol), anhydrous sodium acetate (775 mg) and 5 mL of acetic anhydride. The mixture was refluxed at 150°C for 24 h. After completion of the reaction (by TLC analysis), the reaction mixture was allowed to cool to room temperature. The resulting white solid was collected on a filter, washed with 100 mL of water, and air dried overnight. The white solid was recrystallized by first dissolving in chloroform (10 mL), followed by the addition of 100 mL of ethanol to give pure γ-truxillic anhydride (398 mg, 71% yield) as white crystals: mp 187°C (lit. [30] 190°C); ^1^H NMR (300 MHz, CDCl_3_) δ 7.51–7.39 (m, 4H), 7.37–26 (m, 4H), 7.09–7.06 (m, 2H), 4.34 (t, *J* = 5.7 Hz, 1H) 4.07 (d, *J* = 5.1 Hz, 2H), 3.99 (s, 1H).

#### γ-Truxillic acid 1-naphthyl ester (SB-FI-49)

To a solution of γ-truxillic anhydride (50 mg, 0.18 mmol) and 1-napthol (29 mg, 0.20 mmol) in 1 mL of THF was added diisopropylethylamine (0.03 mL, 0.20 mmol), and the solution was allowed to stir at room temperature for 15 h. After completion of the reaction (by TLC analysis), the reaction mixture was concentrated *in vacuo* to remove all volatiles. The residue was then extracted with 100 mL of dichlorormethane and washed with 100 mL of brine. The organic layer was dried over anhydrous MgSO_4_ and concentrated *in vacuo* to give a brownish solid, which was subsequently purified using flash chromatography on silica gel with 20% ethyl acetate in hexanes followed by 30% ethyl acetate in hexanes as eluents to give γ-truxillic acid 1-naphthyl ester (SB-FI-49, 61 mg, 80% yield) as a white solid: ^1^H NMR (500 MHz, DMSO-*d_6_*) δ 12.27 (b, 1H), 7.87 (d, *J* = 8.5 Hz, 1H), 7.73 (d, *J* = 8.0 Hz, 1H), 7.48–7.24 (m, 13 H), 6.88 (d, *J* = 8.0 Hz, 1H), 6.46 (d, *J* = 7.5 Hz, 1H), 4.59 (t, *J* = 10.5 Hz, 1H), 4.47 (t, *J* = 10.0 Hz, 1H), 4.37 (t, *J* = 10.5 Hz, 1H), 3.85 (t, *J* = 10.5 Hz, 1H). Data are consistent with the literature values. {, 2012 #2890}

### Chemicals in Biological Screening

12-NBD-stearate [12-*N*-methyl-(7-nitrobenz-2-oxa-1,3-diazo)aminostearic acid] was from Avanti Polar Lipids (Alabaster, AL). BMS309403 was from EMD Chemicals (San Diego, CA). Arachidonic acid was from Cayman Chemical (Ann Arbor, MI). 48 virtually screened test compounds from ChemDiv, Inc. (Moscow, Russia). α-Truxillic acid 1-naphthyl ester (SB-FI-26) and γ-truxillic acid 1-naphthyl ester (SB-FI-49) were synthesized at the Institute of Chemical Biology and Drug Discovery, Stony Brook University.

### High throughput fluorescence displacement assay with NBD-stearate

FABP5 was purified and delipidated as described previously [4]. FABP5 (30 µg), NBD-stearate (1 µM), and a competitor test compound were incubated in 30 mM Tris-HCl, 100 mM NaCl buffer (pH 7.6). Competitors included arachidonic acid, BMS309403, 48 test compounds from ChemDiv library, SB-FI-26 and SB-FI-49. The initial assay was run with buffer (30 mM Tris-HCl buffer), negative controls (buffer and NBD-stearate), positive controls (buffer, NBD-stearate, FABP5), and experimental wells with a variable test compound added (arachidonic acid or one of the 48 test compounds) at 10 µM. Test compounds that produced high inhibition and proved statistically significant were then added to the fluorescent assay at 10 µM and tested in triplicate to verify their activity. The most effective test compound and BMS309403 were measured in increasing concentrations (0.01–50 µM), as were the SB-FI-26 and γ-truxillic acid 1-naphthyl ester, which were discovered following the test. The fluorescent assays were tested in the wells of Microtest 96-well Assay Plates, Optilux (BD Biosciences, Franklin Lakes, NJ) and loss of fluorescence intensity was measured with a FLUOstar OPTIMA spectrofluorometer set to excitation and emission wavelengths of 460 nm and 544 nm, respectively. For the most effective test compounds, IC_50_ values were calculated with GraphPad Prism. GraphPad Prism was also used to determine the K_i_ of these select competitors from the equation K_i_ = IC_50_/(1+([NBD-stearate]/K_d_)). The K_d_ of NBD-stearate for FABP5 had been determined previously through incubating FABP5 with increasing concentrations of NBD-stearate. One site binding analysis in GraphPad Prism indicated that the K_d_ of NBD-stearate for FABP5 was 0.16 µM [4].

### Patch-clamp electrophysiology in brain slices

Whole-cell-voltage clamp recordings of dorsal raphe (DR) serotonin (5-HT) neurons were performed as previously described [31]. Briefly, DR neurons were visualized using an upright microscope (BX 51 WI, Olympus, Tokyo, Japan) equipped with a differential interference contrast and infrared imaging system. Somatic recordings from DR neurons were obtained with path electrodes (3–5 mΩ) back-filled with potassium gluconate based internal solution of the following composition: 120 mM potassium gluconate, 10 mM KCl, 10 mM Na_2_-phosphocreatine, 10 mM HEPES, 1 mM MgCl_2_, 1 mM EGTA, 2 mM Na_2_-ATP, 0.25 mM Na-GTP, pH 7.3 (Adjusted with KOH; Osmolarity, 280 to 290 mOsmol/l). All the recordings were conducted in the presence of GABA_A_ receptor antagonist picrotoxin (100 µM). Excitatory postsynaptic currents (EPSCs) were evoked with a single square stimulus (intensity, 1 to 10 V, duration, 100 to 200 µs) delivered via a glass stimulating electrode. EPSCs were amplified with a Multiclamp 700B (Molecular Devices, Union City, CA, USA) and acquired using pClamp 10 software (Molecular Devices).

### Data Analysis

The amplitude of EPSCs was determined by measuring the average current during a 2-ms period at the peak of each EPSC and subtracted from the baseline current determined during a 5-ms time window before the stimulus. All EPSC amplitudes were normalized to the mean baseline amplitude recorded for at least 10 min before drug application. Results in the text and figures are presented as mean ± SEM. Statistical analysis was conducted using the Student's paired t-test.

### AEA uptake

AEA uptake assays in wild-type and FABP5 knockdown HeLa cells were performed exactly as described [4].

### FAAH enzyme assays

Enzyme assays measuring the hydrolysis of [^14^C]AEA in the presence of SB-FI-26 or the FAAH inhibitor URB597 were carried out in HeLa homogenates expressing rat FAAH as described [2].

### PPAR transactivation

PPARα and PPARγ transactivation assays were performed in HeLa cells exactly as described [4]. Briefly, cells were transfected with the PPAR reporter system, incubated with GW7647, rosiglitazone, or SB-FI-26 for 6 hrs, followed by measurement of luciferase and β-galactosidase activity using a luminometer as described [4].

### Animals

We used male C57Bl6 mice (22–30 g, Taconic Farms) for all experiments. The animals were group housed at room temperature and kept on a 12∶12 hour light∶dark cycle with *ad libitum* access to water and food. The animals were habituated to the experimental room for one week before testing.

### Ethics Statement

All experiments were approved by the Stony Brook University Institutional Animal Care and Use Committee (IACUC Permit Number: 2011-1834). The experimenter was blinded to the treatment conditions of each animal.

### Carrageenan-induced paw edema and thermal hyperalgesia

Paw edema was induced by injecting 1% λ-carrageenan (20 µl, in sterile saline) into the plantar surface of the left hind paw and a control solution of saline into the right hind paw using a 27 gauge needle. Paw diameters were measured before carrageenan injection and 4 hours after injection using digital electronic calipers (Fisher) and expressed to the nearest ±0.01 mm. SB-FI-26 (20 mg/kg, i.p.) was dissolved in ethanol∶emulphor∶saline (1∶1∶18), requiring sonication and gentle heating for solubilization, and administered 45 min prior to injection of carrageenan. The cannabinoid receptor antagonists, rimonabant and SR144528 (3 mg/kg, i.p.), in ethanol∶emulphor∶saline (1∶1∶18), were injected 15 min before the FABP inhibitor. Edema is reported as the change in paw diameter at 4 hr over the baseline. Changes in paw diameter of saline-injected contralateral paws were negligible. Thermal hyperalgesia measured the latency to withdraw the paw from a focused beam of radiant heat applied to the plantar surface of the hind paw using a Hargreaves plantar apparatus (Ugo Basile) set at an intensity of 3.0. For each mouse, the average latencies consisted of three trials spaced at least 5 minutes apart. The mice were habituated to the test chamber for 30 min. The cutoff time was set at 30 sec.

### Formalin test

Mice were habituated to the observation chamber (Plexiglas box, 25 cm×25 cm×25 cm) for 30 min prior to formalin injection. The mice subsequently received an injection of formalin (2.5% in saline, 20 µl) into the plantar surface of the right hind paw using a 27 gauge needle. The animals were immediately placed back into the observation chamber and nocifensive behavior (time spent licking or biting the paw) was recorded for 60 min. The formalin test consists of two phases with the first phase (0–5 min) reflecting nociceptor activation and the second phase (15–45 min) reflecting an inflammatory pain response.

### Statistical Analyses

Behavioral data are presented as means ± S.E.M. for the vehicle and inhibitor-treated groups, each consisting of at least 6 animals. Statistical significance between vehicle and inhibitor groups was determined using unpaired t-tests or one-way ANOVA followed by Dunnett's or Tukey-Kramer post hoc analysis. In all cases, differences of p<0.05 were considered significant.
